# Exploring the Clinical Characteristics of COVID-19 Clusters Identified Using Factor Analysis of Mixed Data-Based Cluster Analysis

**DOI:** 10.3389/fmed.2021.644724

**Published:** 2021-07-16

**Authors:** Liang Han, Pan Shen, Jiahui Yan, Yao Huang, Xin Ba, Weiji Lin, Hui Wang, Ying Huang, Kai Qin, Yu Wang, Zhe Chen, Shenghao Tu

**Affiliations:** ^1^Department of Integrated Chinese Traditional and Western Medicine, Tongji Hospital, Tongji Medical College of Huazhong University of Science and Technology, Wuhan, China; ^2^Rehabilitation & Sports Medicine Research Institute of Zhejiang Province, Zhejiang Provincial People's Hospital, People's Hospital of Hangzhou Medical College, Hangzhou, China

**Keywords:** COVID-19, cluster analysis, factor analysis of mixed data, symptoms, laboratory findings, support vector machine

## Abstract

The COVID-19 outbreak has brought great challenges to healthcare resources around the world. Patients with COVID-19 exhibit a broad spectrum of clinical characteristics. In this study, the Factor Analysis of Mixed Data (FAMD)-based cluster analysis was applied to demographic information, laboratory indicators at the time of admission, and symptoms presented before admission. Three COVID-19 clusters with distinct clinical features were identified by FAMD-based cluster analysis. The FAMD-based cluster analysis results indicated that the symptoms of COVID-19 were roughly consistent with the laboratory findings of COVID-19 patients. Furthermore, symptoms for mild patients were atypical. Different hospital stay durations and survival differences among the three clusters were also found, and the more severe the clinical characteristics were, the worse the prognosis. Our aims were to describe COVID-19 clusters with different clinical characteristics, and a classifier model according to the results of FAMD-based cluster analysis was constructed to help provide better individualized treatments for numerous COVID-19 patients in the future.

## Introduction

Over the last year, severe acute respiratory syndrome coronavirus 2 (SARS-CoV-2) has spread all over the world, and it has been concluded that long-term coexistence of humans and the virus is inevitable in the future (1). As a respiratory tract infection disease, coronavirus disease 2019 (COVID-19) usually presents with common symptoms, such as fever, tiredness, headache, cough, and sore throat (2). However, the clinical presentations and disease severity of COVID-19 patients may vary widely. For example, some individuals are asymptomatic, whereas others may develop to life-threatening acute respiratory failure. Although mainly spread via droplets and aerosols, a few SARS-CoV-2-infected individuals show digestive tract symptoms including diarrhea, abdominal pain, nausea, and vomiting, which could be caused by SARS-CoV-2 infection of the digestive tract system or triggered by therapeutic drugs, liver function injury and mental factors (3–6). In addition to specific symptoms, many publications have revealed that the disease severity and prognosis can be predicted by lymphocytes, D-dimer, C-reactive protein (CRP), and other laboratory indicators (7–10).

Previously, the clinical classifications of COVID-19 were mainly based on clinical indexes and radiological manifestations (11, 12). However, the potential relationship among disease severity, syndromes and laboratory tests was barely considered in those classifications. Therefore, it is necessary and meaningful to consider those relationships comprehensively and identify the subtypes of COVID-19.

Our study aimed to identify the subtypes of COVID-19 by using an unsupervised classifier. Factor analysis of mixed data (FAMD)-based cluster analysis was used to identify COVID-19 subtypes based on clinical symptoms, laboratory tests and demographic characteristics (13). Three COVID-19 subtypes were identified in our study, and the differences among the COVID-19 subtypes would contribute to our understanding of COVID-19 clinical characteristics. Moreover, subtypes with different clinical characteristics in this study showed different prognoses. Given this, a support vector machine (SVM)-based classifier was trained to recognize different COVID-19 subgroups. We believe that this classifier model could assist clinicians in rapidly identifying individuals with more severe and worse prognoses according to their symptoms and laboratory findings.

## Materials and Methods

### Participants

**Figure 1:**
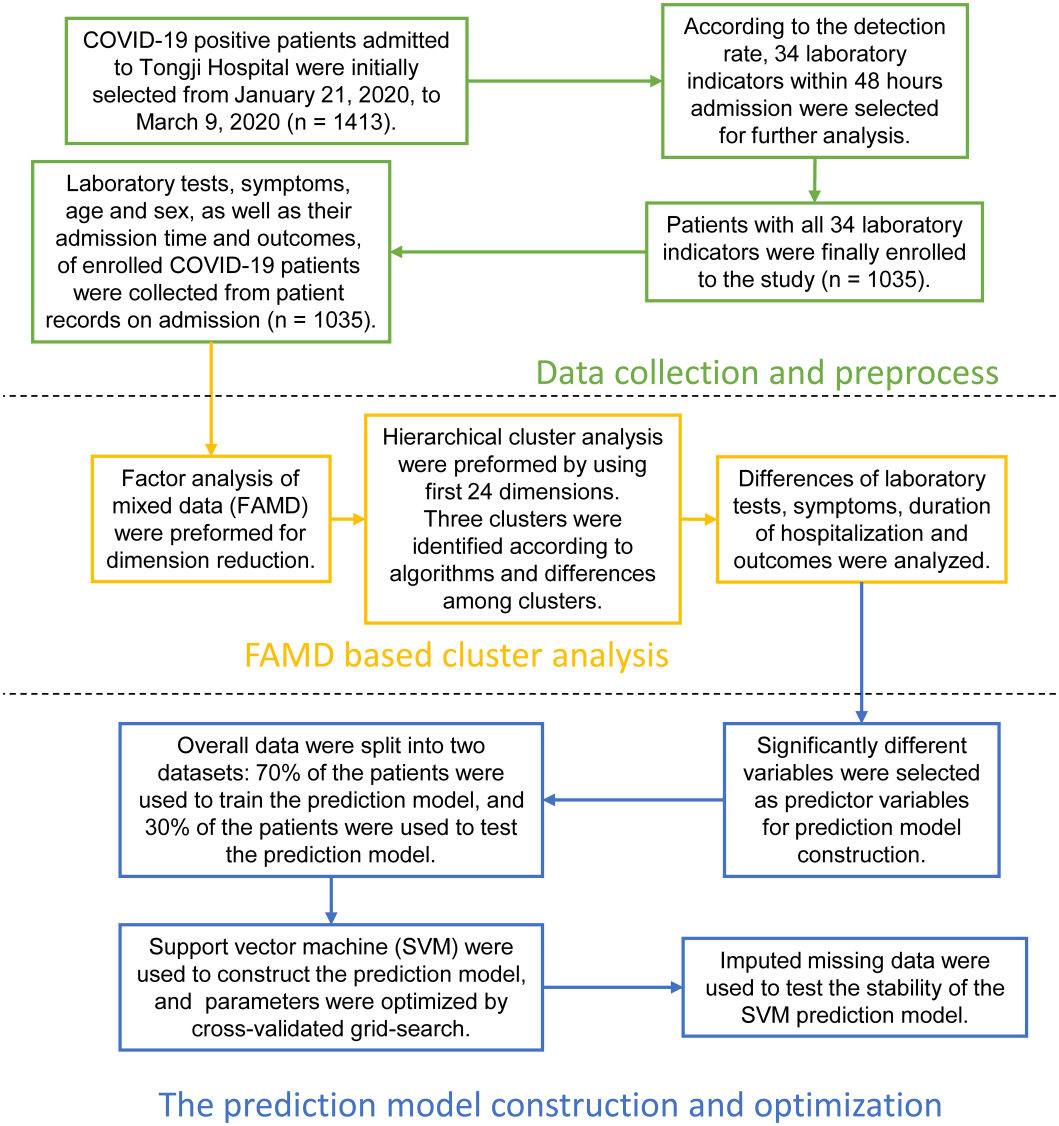
Overall design and analysis process of the study. The study was organized into three parts. First section: Data collection and preprocess. COVID-19 positive patients admitted to Tongji Hospital of Tongji Medical College of Huazhong University of Science and Technology were initially selected from January 21, 2020, to March 9, 2020. Their demographic information, laboratory indicators within 48 h admission, medical record-based admission symptoms, as well as duration of hospitalization and outcomes were collected. Afterwards, we investigated the rates of missing laboratory data, and 34 laboratory indicators tested by most patients were identified. Patients with missing 34 laboratory indicators were culled. Laboratory tests, symptoms, duration of hospitalization, and outcomes of 1,035 COVID-19 patients were finally included in the study. Second section: Factor analysis of mixed data (FAMD) based cluster analysis. We used FAMD as the dimensionality reduction method, and first 24 dimensions were selected and retained for further unsupervised cluster analysis. Three major clusters were identified according to algorithms and differences among clusters. We compared the differences in laboratory tests, symptoms, duration of hospitalization, and outcomes among the three clusters in detail. Third section: Prediction model construction and optimization. We constructed a COVID-19 support vector machine (SVM) classifier according to the results of the unsupervised clustering in this section. Firstly, overall data was randomly divided into 70% training and 30% test dataset. The optimal parameters were found by using the grid search based on 10-fold cross validation on 70% training dataset, and the classification performance of the model were tested by testing 30% dataset. Secondly, all patients were randomly divided into 50% training and 50% missing test dataset. About 1–10% laboratory data in the missing dataset were removed randomly, and missing data were imputed by *k*-nearest neighbor (KNN) method. The SVM model was trained by 50% training dataset, and missing test dataset was used to test the performance of the SVM model in the case of missing data.

Inpatient COVID-19 patients from January 21, 2020 to March 9, 2020 were initially recruited, and their medical history and laboratory findings were collected from Tongji Hospital of Tongji Medical College of Huazhong University of Science and Technology. Ethics approval was obtained by the ethics committee of Tongji Hospital of Tongji Medical College of Huazhong University of Science and Technology, and the approval reference number is TJ-IRB20200365. An exemption was granted obtaining written informed consent from the subjects. The present study design is depicted in Figure 1.

### Data Extraction

We selected the above mentioned 1,413 COVID-19 patients with positive COVID-19 nucleic acid or antibodies for this study, which were tested either before admission or during admission. Patients with incomplete medical records were excluded, and the remaining individuals' admission records were analyzed and collected by two clinicians independently to determine their age, sex, and symptoms because of the unstructured nature of the medical records. Simultaneously, laboratory data within 48 h after admission, including routine blood tests, blood biochemistry, coagulation function and other laboratory indicators, were also screened. However, laboratory tests performed at the time of patient admission to the hospital are not always the same but depend on the severity of each patient condition. Nevertheless, some tests such as routine blood tests, were analyzed for almost all patients within 48 h, but other tests such as interleukin tests, were performed only for severely ill patients. Consequently, we balanced the selection of patients and laboratory indicators to ensure that as many patients and indicators were included in the study as possible. For this purpose, we examined the missing rate of each laboratory indicator (Supplementary Figures 1, 2). Finally, only tests with more than 90% completeness rates were selected for further analysis, and COVID-19 patient with missing laboratory were also excluded. Symptoms, laboratory indicators, age, sex, were finally collected and analyzed in our study (Tables 1–3). Total hospital days and outcomes were also collected to compare in-hospital survival rate, which was also the endpoint of this study (Table 1).

**Table 1:** Demographic characteristics, hospitalization days, and outcomes of 1,035 COVID-19 patients.

**Characteristics**		**Median (IQR)**
Age (years)		63.20 (52.00–70.33)
Sex	Female	525 (50.72%)
	Male	510 (49.28%)
Hospitalization days (days)		21.00 (14.00–31.00)
Outcomes	Dead	61 (5.89%)
	Alive	974 (94.1%)

*Continuous variables are presented as median (interquartile ranges), while categorical variables as counts and percentages (%)*.

**Table 2:** Laboratory findings within 48 h after admission of 1,035 COVID-19 patients.

**Laboratory tests**	**Median (IQR)**	**Reference intervals**
ALT (U/L)	23.00 (15.00–40.00)	≤ 41
AST (U/L)	24.00 (18.00–36.00)	≤ 40
γ-GT (U/L)	29.00 (18.00–52.00)	10–71
Albumin (g/L)	36.10 (32.40–39.90)	35–52
Globulin (g/L)	31.80 (28.50–35.60)	20–35
Total protein (g/L)	68.30 (64.90–72.00)	64–83
Creatinine (μmol/L)	68.00 (57.00–83.00)	59–104
Urea (mmol/L)	4.40 (3.50–5.70)	3.1–8.0
Uric acid (μmol/L)	262.10 (208.00–324.40)	202.3–416.5
Total cholesterol (mmol/L)	3.82 (3.23–4.48)	<5.18
Blood glucose (mmol/L)	5.80 (5.11–7.31)	4.11–6.05
LDH (U/L)	249.00 (200.00–316.00)	135–225
ALP (U/L)	67.00 (55.00–81.00)	40–130
WBC count (× 10^9^/L)	5.78 (4.63–7.26)	3.50–9.50
RBC count (× 10^12^/L)	4.09 (3.69–4.47)	4.30–5.80
Lymphocyte rate (%)	22.40 (14.60–30.50)	20–50
Lymphocyte count (× 10^9^/L)	1.24 (0.85–1.65)	1.10–3.20
Monocyte rate (%)	8.50 (6.80–10.30)	3.0–10.0
Monocyte count (× 10^9^/L)	0.49 (0.37–0.64)	0.10–0.60
Neutrophil rate (%)	66.30 (57.30–75.70)	40.0–75.0
Neutrophil count (× 10^9^/L)	3.72 (2.74–5.23)	1.80–6.30
Eosinophil rate (%)	1.00 (0.20–2.00)	0.4–0.8
Eosinophil count (× 10^9^/L)	0.06 (0.01–0.12)	0.02–0.52
Basophil rate (%)	0.20 (0.10–0.40)	0.0–1.0
Basophil count (× 10^9^/L)	0.01 (0.01–0.03)	0.00–0.10
Hematocrit (%)	36.60 (33.30–39.40)	40.0–50.0
Hemoglobin (g/L)	126.00 (115.00–136.00)	130.0–175.0
Platelet (× 10^9^/L)	237.00 (180.00–309.50)	125.0–350.0
D-dimer (μg/ml FEU)	0.67 (0.34–1.48)	<0.5
PTA (%)	93.00 (86.00–101.00)	75.0–125.0
PT (s)	13.70 (13.10–14.20)	11.5–14.5
INR	1.05 (0.99–1.10)	0.80–1.20
CRP (mg/L)	10.20 (1.90–49.50)	<1
eGFR (ml/min/1.73 m^2^)	92.60 (78.80–102.50)	>90

*ALT, alanine transaminase; AST, aspartate transaminase; γ-GT, gamma-glutamyl transferase; LDH, lactic dehydrogenase; ALP, alkaline phosphatase; WBC, white blood cell; RBC, red blood cell; PTA, prothrombin time activity; PT, prothrombin time; INR, international normalized ratio; CRP, C-reactive protein; eGFR, estimated glomerular filtration rate. The reference intervals of laboratory tests were aligned with those used by the laboratory of Tongji Hospital. Continuous variables are presented as median (interquartile ranges)*.

**Table 3:** Frequencies of symptoms before admission of 1,035 COVID-19 patients.

**Symptoms**	***n* = 1,035 (%)**
Fever	797 (77.00%)
Chills	196 (18.94%)
Inappetence	301 (29.08%)
Fatigue	334 (32.27%)
Myalgia	171 (16.52%)
Headache	79 (7.63%)
Palpitations	47 (4.54%)
Night sweat	40 (3.86%)
Dizziness	50 (4.83%)
Cough	774 (74.78%)
Nasal obstruction or runny nose	28 (2.70%)
Sore throat	63 (6.09%)
Dyspnea	361 (34.88%)
Diarrhea	245 (23.67%)
Abdominal pain	20 (1.93%)
Nausea	108 (10.43%)
Vomiting	56 (5.41%)

*Categorical variables are presented as counts and percentages (%)*.

### Identification of COVID-19 Clusters

Both many studies and clinical experience indicate potential links between different laboratory indicators and symptoms among COVID-19 patients. There are also correlations between some laboratory tests, for example, lymphocyte count, and percentage of lymphocytes. Thus, factor analysis of mixed data (FAMD), a principal component method dedicated to analyzing a data set containing both quantitative and qualitative variables, was used to deconstruct the original complex data into fewer relevant factors. FAMD was performed using the R package FactoMineR (https://cran.r-project.org/package=FactoMineR), and the factoextra package (https://cran.r-project.org/package=factoextra) was used to extract the FAMD results. The first 24 dimensions were selected and retained for further cluster analysis, as these explained >80% of the total variance.

Cluster analysis is one of the most popular unsupervised learning methods to identify subgroups sharing similar characteristics, with no predefined information necessary. Agglomerative hierarchical cluster analysis of COVID-19 patients based on the FAMD-transformed matrix was performed according to the Ward criterion, which could minimize the total intracluster variance. Function dist() and function hclust() from the R package base were used for the cluster analysis. The R packages ggtree (https://cran.r-project.org/package=ggtree) and ape (https://cran.r-project.org/package=ape) were used to visualize the cluster analysis result, and the last several steps of cluster analysis were shown as a dendrogram, which was constructed by the R packages ggraph (https://cran.r-project.org/package=ggraph) and tidygraph (https://cran.r-project.org/package=tidygraph) (14–16). The R package NbClust was used to evaluate the range of the number of COVID-19 patients (17).

### Difference in Prognosis Among COVID-19 Clusters

Considering significantly different characteristics in different COVID-19 clusters, we assume that they have distinct prognoses. Thus, the prognoses of COVID-19 patients were recorded as the hospitalization days and outcomes. Patient outcomes were followed up until discharge from the hospital or death. Total hospital days were compared, survival analysis was performed using the R package survival (https://cran.r-project.org/package=survival), and Kaplan-Meier survival curves were plotted by the R package survminer (https://cran.r-project.org/package=survminer) (18).

### Statistical Analysis

Statistical analyses were conducted in R (R version 3.6.0). Continuous data are expressed as medians (interquartile range), and the rate is expressed as counts (percentages). Normal distribution and homogeneous variance were tested for all data. Normal distribution test was performed by Shapiro–Wilk test via function shapiro.test() in R, and homogeneity of variance test was performed by Bartlett's Test via function bartlett.test() in R. Differences in characteristics between the clusters were assessed using analysis of variance for continuous normally distributed and homogeneous variance values, and the nonparametric Kruskal–Wallis test with Dunn's posttest for continuous nonnormally distributed and/or inhomogeneous variances values using the R package FSA (https://cran.r-project.org/package=FSA). The difference between rates was tested by χ^2^ test or Fisher's exact test for categorical variables. Survival curves were compared by log-rank analysis. The Benjamini–Hochberg procedure was used for multiple comparison correlation. A *p* < 0.05 was considered statistically significant. Box plots and radar charts were compiled using the R packages ggpubr (https://cran.r-project.org/package=ggpubr) and fmsb (https://cran.r-project.org/package=fmsb), respectively.

### Construction of the Classifier Model to Forecast COVID-19 Clusters

SVM is a popular supervised learning method that constructs hyperplanes in a high-dimensional space to separate training data into different classes and is often used for classification. In our study, an SVM classifier model of COVID-19 clusters were constructed by the R package e1071 (https://cran.r-project.org/package=e1071). Indicators of the COVID-19 patients on admission, including their clinical symptoms and laboratory tests, with statistically significant differences among the three clusters, were chosen as the predictor variables, and the response variable was the FAMD-based clustering results.

All 1,035 patients were randomly divided into 70% training and 30% test datasets. We implemented a grid search and 10-fold cross validation for tuning and validating the prediction model on the training dataset. Then the model with optimal parameters were tested on the test dataset. Kappa statistic was calculated using the R package caret (https://CRAN.R-project.org/package=caret) and used to evaluate the performance of SVM model with different kernel and parameters. A receiver operating characteristic (ROC) curve was constructed, and the ROC areas under the curve (AUCs) were calculated using the R package pROC (https://cran.r-project.org/package=pROC) (19).

To test the performance of model in the case of missing data, all patients were divided into 50% training and 50% missing test dataset. About 1–10% laboratory data in the missing dataset was removed randomly using the R package simFrame (https://cran.r-project.org/package=simFrame) (20), and missing data were imputed by k-nearest neighbor (KNN) method using the R package DMwR2 (https://cran.r-project.org/package=DMwR2). The SVM model was firstly trained on the 50% training dataset and then tested on the 50% missing dataset. Tests were repeated 50 times with the same missing rate.

## Results

### Demographic, Clinical, and Laboratory Characteristics of 1,035 COVID-19 Patients

A total of 1,413 COVID-19 positive patients were primarily enrolled to the study. The heat map of missing laboratory tests analysis was illustrated in Supplementary Figure 1, and the completeness rates of laboratory tests were illustrated in Supplementary Figure 2. Only the laboratory tests with more than 90% completeness were kept for the next analysis. In addition, to ensure the accuracy of the study, only patients with all more than 90% completeness laboratory tests were retained. After the screening process, 1,035 patients and 34 laboratory indicators remained. Then we collected age, sex, syndromes, and laboratory findings from 1,035 COVID-19 patients. Women made up 50.72%, and the median age of the group was 63.20 (52.00–70.33). The most common clinical symptom was fever (77.00%), followed by cough (74.78%), dyspnea (34.88%), fatigue (32.27%), and inappetence (29.08%). Lactate dehydrogenase (LDH), albumin, blood glucose, red blood cell (RBC) count, lymphocyte count, percentage of lymphocytes, percentage of eosinophils, hemoglobin, hematocrit, estimated glomerular filtration rate (eGFR), CRP, and D-dimer were clearly abnormal in all 1,035 individuals.

### FAMD-Based Cluster Analysis

**Figure 2:**
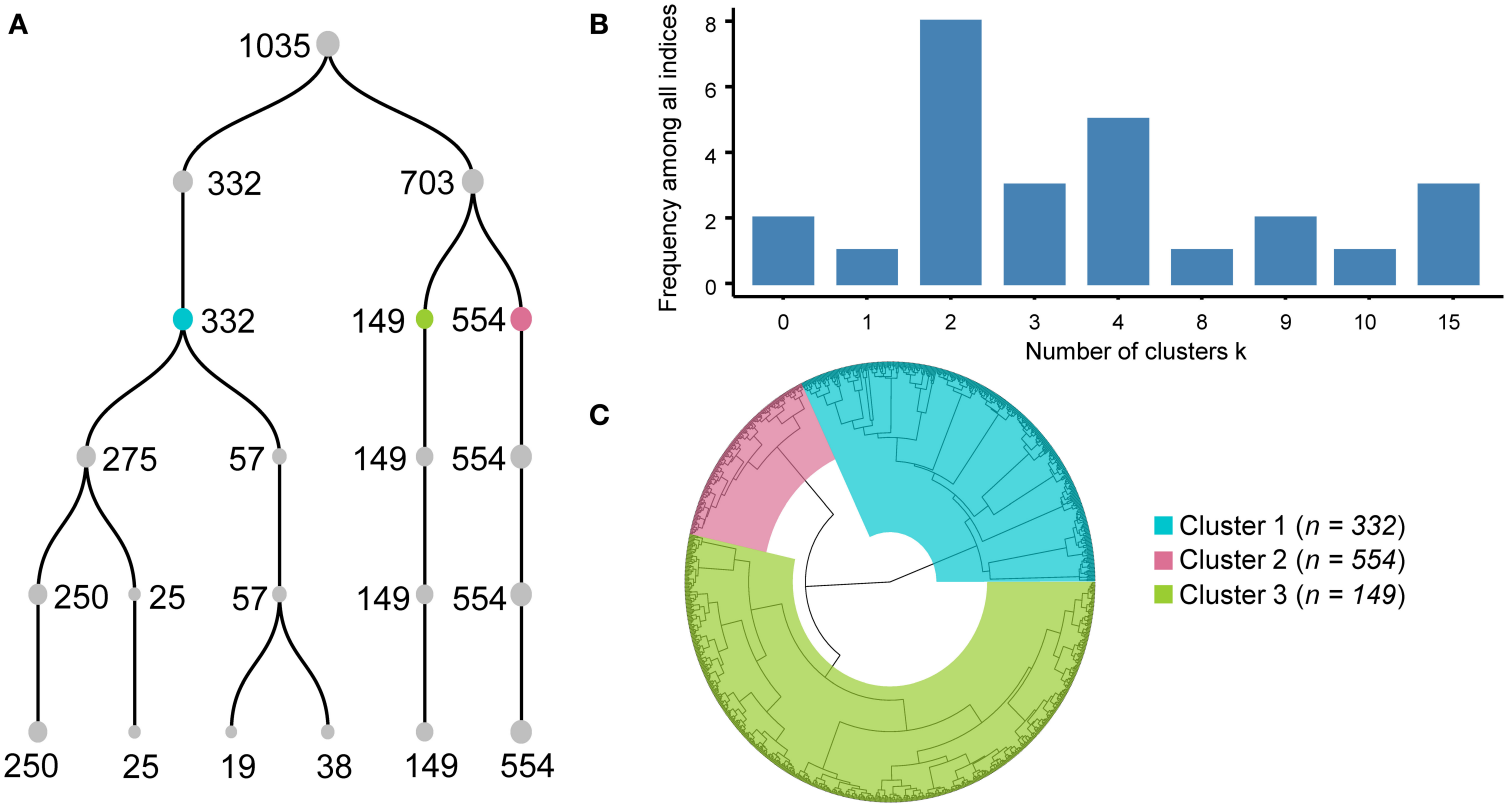
Processes and results of factor analysis of mixed data-based cluster analysis. Dendrogram **(A)** shows the processes of combination from six clusters to one cluster. Each number near the point means the number of individuals in each cluster. Histogram **(B)** illustrated that different algorithm in R package NbClust supported different clustering scheme, and dividing into two, three or four clusters were endorsed by the majority of algorithms. Circular dendrogram **(C)** shows the total processes of cluster analysis. Cluster results are colored blue for Cluster A, green for Cluster B, and red for Cluster C.

FAMD was applied to the original matrix, which consisted of age, sex, 17 symptoms and 34 laboratory indicators of the 1,035 COVID-19 patients, and 53 dimensions were obtained (Supplementary Table 1). Variances of 53 dimensions decreased gradually, and variances of the top 24 dimensions accounted for more than 80% of the total variance. Thus, the top 24 dimensions were retained for further analysis. Subsequently, unsupervised hierarchical cluster analysis was performed with the matrix made with the top 24 dimensions values of 1,035 individuals. A dendrogram (Figure 2A) showed the last five steps of cluster analysis.

Agglomerative hierarchical cluster analysis had a bottom-up approach, and all subjects were clustered into a single cluster at last, so it had to decide when to stop clustering. If the number of clusters was too small, the clinical features of COVID-19 patients would be more homogeneous, and it could not well-reveal the clinical pattern of COVID-19. Conversely, the COVID-19 patterns represented by multiple clusters were unintelligible and difficult to understand. Therefore, it was crucial to determine how many clusters to use. We first evaluated the range of the number of clusters by the R package NbClust using 26 algorithms. Six, four, and six algorithms supposed that the best cluster numbers were 2, 3, and 4, respectively (Figure 2B), which indicated that the range of best cluster number was 2–4.

**Figure 3:**
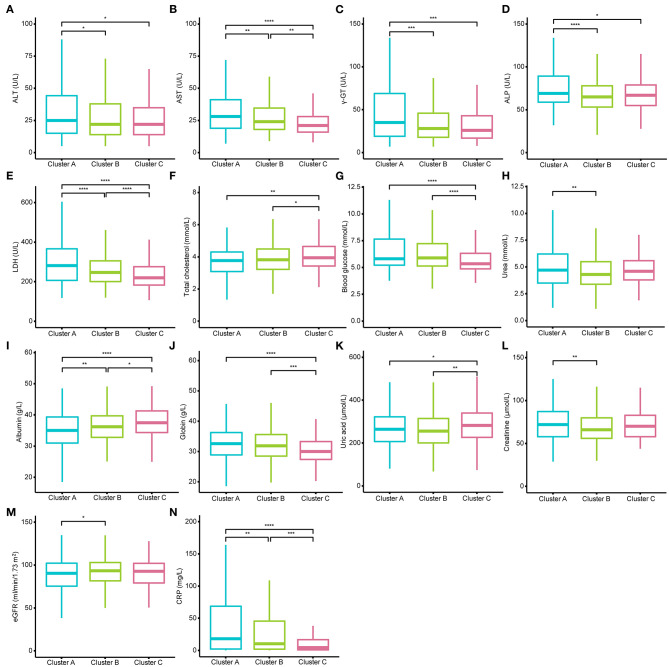
Different levels of blood biochemistry tests among the three clusters. **(A–N)** show the different levels of alanine transaminase (ALT), aspartate transaminase (AST), gamma-glutamyl transferase (γ-GT), alkaline phosphatase (ALP), lactic dehydrogenase (LDH), total cholesterol, blood glucose, urea, albumin, globin, uric acid, creatinine, estimated glomerular filtration rate (eGFR), and C-reactive protein (CRP) among the three clusters. AST, LDH, CRP, and albumin could well-distinguish the three clusters. The Kruskal–Wallis tests with Dunn's post-test was performed, and the *p-*value were adjusted by the Benjamini–Hochberg procedure. *p < 0.05; **p < 0.01; ***p < 0.001; ****p < 0.0001.

**Figure 4:**
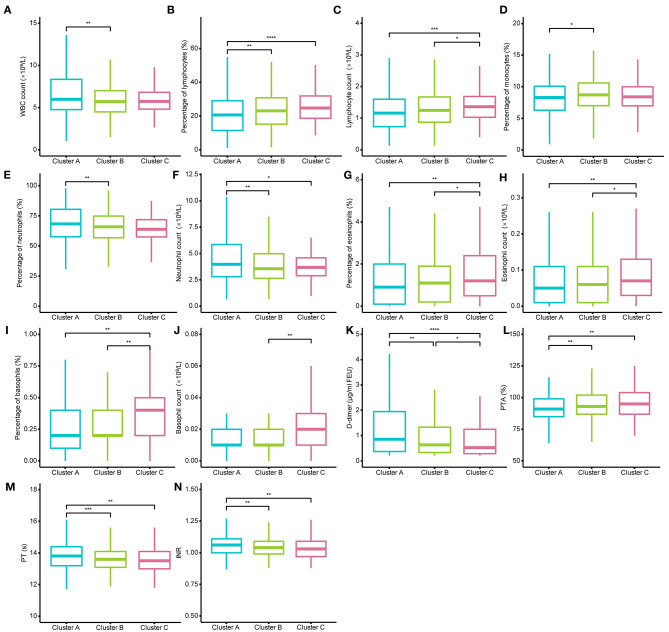
Different levels of routine blood tests and coagulation function among the three clusters. **(A–N)** show the different levels of white blood cell (WBC) count, percentage of lymphocytes, lymphocyte count, percentage of monocytes, percentage of neutrophils, neutrophil count, percentage of eosinophils, eosinophil count, percentage of basophils, basophil count, D-dimer, prothrombin time activity (PTA), and prothrombin time (PT), international normalized ratio (INR). D-dimer could well distinguish the three clusters. The Kruskal–Wallis tests with Dunn's posttest was performed, and *p*-values were adjusted by the Benjamini–Hochberg procedure. *p < 0.05; **p < 0.01; ***p < 0.001; ****p < 0.0001.

Then, we examined the differences in laboratory tests among different clusters under the conditions of dividing them into 2, 3, and 4 clusters separately. All laboratory test values under the conditions of dividing them into 2, 3, and 4 clusters did not meet normal distribution and homogeneity of variance, so the nonparametric Kruskal–Wallis test with Dunn's posttest was used for multiple comparison analysis (Supplementary Tables 2–5). We found that when the individuals were divided into two clusters, it was not hard to observe that almost all indexes of patients in Cluster A were more severe than those in patients of Cluster B (Supplementary Figure 3). When the COVID-19 individuals were divided into four clusters, the levels of CRP, D-dimer, PT, and the percentage of lymphocytes, which have been reported as crucial disease severity indexes, had no differences between Cluster D and the other three clusters (Supplementary Figure 4). In contrast, the above crucial indicators can be distinguished well when divided into three clusters (Figures 3, 4). Thus, it is natural to suppose that the severity of COVID-19 patients in Cluster D had no significant difference, which indicated that this clustering scheme might just be in accordance with the characteristics of the data itself instead of the clinical phenotypes of COVID-19. Accordingly, we thought that dividing into three clusters was the best clustering scheme, and the 1,035 COVID-19 patients were divided into three clusters in the following analysis (Figure 2C).

### Demographic, Symptoms, and Laboratory Characteristics in Different COVID-19 Clusters

**Figure 5:**
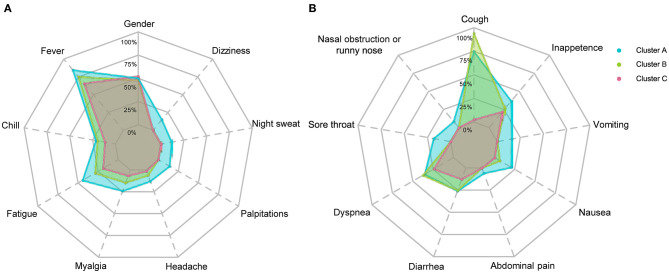
Frequencies of symptoms of COVID-19 patients in three clusters. Frequencies of systematic symptoms and nervous system symptoms are shown in **(A)** and frequencies of respiratory symptoms and digestive tract symptoms are shown in **(B)**. Symptoms labeled with dashed lines have significant differences (*p* < 0.05) among the three clusters. The χ^2^ test or Fisher's exact test were performed.

The demographic characteristics of the three clusters are presented in Table 4. Surprisingly, there was no difference in age or sex among the clusters. The laboratory indicators and syndrome characteristics of the three clusters are shown in Tables 5, 6. Most laboratory findings and syndromes differed among the clusters, and the differences in laboratory indicators and syndromes among the three clusters can be seen intuitively from box plots (Figures 3, 4) and radar charts (Figure 5). Overall, the patients in Cluster A presented with the most severe conditions at the time of admission, and patients in Cluster C were the mildest. In contrast, the conditions of individuals in Cluster B were in between these two.

**Table 4:** Demographic characteristics of three COVID-19 clusters identified by Factor Analysis of Mixed Data-based cluster analysis.

**Characteristics**		**Cluster A (*n* = 332)**	**Cluster B (*n* = 554)**	**Cluster C (*n* = 149)**	***p*-value**
Age (years)		63.28 (51.95– 71.15)	63.23 (51.62–69.67)	62.86 (54.18–70.98)	0.81651
Sex	Female	165	289	71	0.55872
	Male	167	265	78	
Hospitalization days (days)		23.00 (14.00–35.25)	22.00 (14.00–32.00)	19.00 (11.00–24.00)	<0.00001
Outcomes	Dead	33	25	3	0.00052
	Alive	299	529	146	

*Continuous variables are presented as median (interquartile ranges), and Kruskal–Wallis test was applied for continuous variables. Categorical variables are expressed as counts and percentages (%), and the χ^2^ test or Fisher's exact test were applied for categorical variables*.

**Table 5:** Laboratory findings of three COVID-19 clusters identified by Factor Analysis of Mixed Data-based cluster analysis.

**Laboratory tests**	**Cluster A (*n* = 332)**	**Cluster B (*n* = 554)**	**Cluster C (*n* = 149)**	***p-*value**
ALT (U/L)	25.00 (15.00–44.25)	22.00 (14.00–38.00)	22.00 (14.00–35.00)	0.00294
AST (U/L)	28.00 (19.00–41.25)	24.00 (18.00–34.75)	21.00 (16.00–28.00)	<0.00001
γ-GT (U/L)	35.00 (19.00–69.00)	28.00 (18.00–46.00)	26.00 (17.00–43.00)	0.00002
Albumin (g/L)	35.05 (31.00–39.40)	36.20 (32.83–39.78)	37.50 (34.40–41.30)	0.00010
Globulin (g/L)	32.60 (28.90–36.30)	31.90 (28.53–35.65)	30.00 (27.40–33.30)	0.00006
Total protein (g/L)	67.90 (64.85–71.73)	68.70 (64.90–72.30)	68.50 (64.90–71.50)	0.44564
Creatinine (μmol/L)	72.00 (58.00–87.25)	66.00 (56.00–80.00)	70.00 (58.00–83.00)	0.00361
Urea (mmol/L)	4.70 (3.50–6.23)	4.30 (3.40–5.50)	4.60 (3.80–5.60)	0.01137
Uric acid (μmol/L)	264.20 (207.53–322.53)	255.65 (200.95–315.00)	282.00 (226.70–340.00)	0.00556
Total cholesterol (mmol/L)	3.77 (3.09–4.31)	3.82 (3.23–4.50)	3.94 (3.44–4.65)	0.00162
Blood glucose (mmol/L)	5.81 (5.22–7.65)	5.89 (5.14–7.24)	5.34 (4.88–6.33)	0.00011
LDH (U/L)	281.00 (207.00–366.25)	247.00 (201.25–305.75)	220.00 (183.00–276.00)	<0.00001
ALP (U/L)	69.00 (59.00–89.25)	65.00 (53.25–78.00)	67.00 (55.00–79.00)	0.00017
WBC count (× 10^9^/L)	5.98 (4.77–8.35)	5.71 (4.49–7.02)	5.72 (4.83–6.81)	0.00722
RBC count (× 10^12^/L)	4.11 (3.64–4.47)	4.07 (3.70–4.46)	4.11 (3.73–4.47)	0.86039
Lymphocyte rate (%)	20.65 (11.48–29.10)	23.05 (15.20–30.90)	24.70 (18.70–31.90)	0.00023
Lymphocyte count (× 10^9^/L)	1.16 (0.73–1.60)	1.25 (0.87–1.67)	1.36 (1.03–1.69)	0.00316
Monocyte rate (%)	8.30 (6.30–10.10)	8.70 (7.03–10.60)	8.40 (7.00–10.00)	0.03766
Monocyte count (× 10^9^/L)	0.49 (0.37–0.65)	0.48 (0.37–0.64)	0.50 (0.38–0.60)	0.80982
Neutrophil rate (%)	68.40 (57.68–80.53)	65.95 (56.83–74.90)	63.80 (57.60–71.90)	0.00332
Neutrophil count (× 10^9^/L)	3.98 (2.80–5.87)	3.55 (2.65–4.99)	3.68 (2.90–4.60)	0.00318
Eosinophil rate (%)	0.90 (0.10–2.00)	1.10 (0.20–1.90)	1.20 (0.50–2.40)	0.03027
Eosinophil count (× 10^9^/L)	0.05 (0.01–0.11)	0.06 (0.01–0.11)	0.07 (0.03–0.13)	0.05311
Basophil rate (%)	0.20 (0.10–0.40)	0.20 (0.20–0.40)	0.40 (0.20–0.50)	0.00175
Basophil count (× 10^9^/L)	0.01 (0.01–0.02)	0.01 (0.01–0.02)	0.02 (0.01–0.03)	0.01233
Hematocrit (%)	36.60 (32.60–39.43)	36.45 (33.40–39.30)	36.90 (33.60–39.30)	0.61877
Hemoglobin (g/L)	126.00 (113.00–136.00)	125.00 (115.00–136.00)	127.00 (115.00–137.00)	0.89523
Platelet (× 10^9^/L)	239.50 (179.00–301.00)	235.00 (180.25–314.00)	235.00 (182.00–304.00)	0.94041
D-dimer (μg/ml FEU)	0.86 (0.37–1.95)	0.63 (0.33–1.33)	0.52 (0.29–1.25)	0.00008
PTA (%)	91.00 (85.00–99.00)	93.00 (87.00–102.00)	95.00 (87.00–104.00)	0.00179
PT (s)	13.80 (13.20–14.40)	13.60 (13.10–14.10)	13.50 (13.00–14.10)	0.00096
INR	1.06 (1.00–1.11)	1.04 (0.99–1.09)	1.03 (0.97–1.09)	0.00133
CRP (mg/L)	18.05 (2.28–68.88)	10.25 (2.10–45.70)	4.30 (1.00–16.90)	<0.00001
eGFR (ml/min/1.73 m^2^)	90.4 (75.45–102.20)	93.35 (81.83–103.05)	92.60 (79.20–102.00)	0.04890

*ALT, alanine transaminase; AST, aspartate transaminase; γ-GT, gamma-glutamyl transferase; LDH, lactic dehydrogenase; ALP, alkaline phosphatase; WBC, white blood cell; RBC, red blood cell; PTA, prothrombin time activity; PT, prothrombin time; INR, international normalized ratio; CRP, C-reactive protein; eGFR, estimated glomerular filtration rate. Laboratory tests are presented as median (interquartile ranges). Comparisons were performed using the Kruskal–Wallis test*.

**Table 6:** Frequencies of symptoms of three COVID-19 clusters identified by Factor Analysis of Mixed Data-based cluster analysis.

**Symptoms**	**Cluster A (%) (*n* = 332)**	**Cluster B (%) (*n* = 554)**	**Cluster C (%) (*n* = 149)**	***p*-value**
Fever	281 (84.64%)	418 (75.45%)	98 (65.77%)	0.00001
Chills	71 (21.39%)	108 (19.49%)	17 (11.40%)	0.02649
Inappetence	122 (36.75%)	146 (26.35%)	33 (22.15%)	0.00067
Fatigue	147 (44.28%)	159 (28.70%)	28 (18.79%)	<0.00001
Myalgia	80 (24.10%)	81 (14.62%)	10 (6.71%)	<0.00001
Headache	43 (12.95%)	34 (6.14%)	2 (1.34%)	<0.00001
Palpitations	47 (14.16%)	0 (0.00%)	0 (0.00%)	<0.00001
Night sweat	39 (11.75%)	1 (0.18%)	0 (0.00%)	<0.00001
Dizziness	49 (14.76%)	1 (0.18%)	0 (0.00%)	<0.00001
Cough	249 (75.00%)	521 (94.04%)	4 (2.68%)	<0.00001
Nasal obstruction or runny nose	28 (8.43%)	0 (0.00%)	0 (0.00%)	<0.00001
Sore throat	61 (18.37%)	2 (0.36%)	0 (0.00%)	<0.00001
Dyspnea	117 (35.24%)	208 (37.55%)	36 (24.16%)	0.00832
Diarrhea	86 (25.90%)	140 (25.27%)	19 (12.75%)	0.00188
Abdominal pain	20 (6.02%)	0 (0.00%)	0 (0.00%)	<0.00001
Nausea	69 (20.78%)	38 (6.86%)	1 (0.67%)	<0.00001
Vomiting	53 (15.96%)	3 (5.42%)	0 (0.00%)	<0.00001

*Symptoms are presented as counts and percentages (%). Comparisons were performed using the χ^2^ test or Fisher's exact test*.

A total of 332 patients were included in Cluster A. Almost all laboratory indicators and symptoms were worst in Cluster A. In terms of blood biochemistry tests, patients in Cluster A presented the highest levels of alanine transaminase (ALT), aspartate transaminase (AST), gamma-glutamyl transferase (γ-GT), LDH, alkaline phosphatase (ALP), total cholesterol, blood glucose, and albumin and the lowest level of globin. Additionally, their median eGFR was abnormally low and the level of creatinine was high in Cluster A. In routine blood tests, Cluster A showed higher white blood cell (WBC) counts, neutrophil counts, percentage of neutrophils, and lower levels of lymphocyte counts and percentage of lymphocytes than the other two clusters. Moreover, the levels of eosinophil and basophils were also lowest in Cluster A individuals. Regarding coagulation function, Cluster A patients exhibited higher levels of D-dimer, prothrombin time (PT), and international normalized ratio (INR) and lower levels of prothrombin time activity (PTA). Finally, the highest level of CRP was also observed in Cluster A, and the median CRP was up to 18.05 mg/L.

The frequencies of many systemic and neurological symptoms in Cluster A patients, including fever, chills, fatigue, myalgia, headache, palpitation, night sweat, and dizziness, were highest among the three clusters. For respiratory symptoms and digestive tract symptoms, the frequencies of nasal obstruction or runny nose, sore throat, dyspnea, diarrhea, abdominal pain, nasal obstruction or runny nose, vomiting and anorexia were also at the top level among the three clusters. Although the frequency of cough was the second highest among the clusters, three-quarters of individuals in Cluster A had cough before their hospitalization. Therefore, Cluster A could also be designated as a severe cluster.

Cluster B was the largest cluster in this study, and almost all of their laboratory indicators and frequencies of symptoms seem to be intermediate between Clusters A and C; however, the frequency of cough was an exception. The most prominent symptom in Cluster B was a cough, which was reported in almost all individuals in Cluster B. In addition to a cough, patients in Cluster B showed moderate frequencies of fever, fatigue, myalgia, headache and nausea. The frequencies of chills, dyspnea and diarrhea in Cluster B were as high as those in Cluster A; however, the frequencies of palpitation, night sweat, dizziness, nasal obstruction or runny nose, sore throat, abdominal pain, vomiting, and anorexia in Cluster B were uniformly low relative to those in Cluster C. Notably, the frequencies of palpitation, night sweat, dizziness, nasal obstruction or runny nose, sore throat, abdominal pain, nausea, and vomiting in Clusters B and C were very close to 0%.

Cluster C, with 149 individuals, had the lowest number of COVID-19 patients and the lowest levels of almost all indicators, including CRP and symptoms, among the three clusters. It is worth mentioning that the conditions of individuals in Cluster C were rather mild, not only because of those better indicators but also because of their close to 0% frequencies of palpitations, night sweats, dizziness, nasal obstruction or runny nose, sore throat, abdominal pain, nausea and vomiting, and even coughing. In contrast, the frequencies of fever (65.77%), dyspnea (24.16%), anorexia (22.15%), fatigue (18.79%), diarrhea (12.75%), and chills (11.40%) in Cluster C were relatively high, but they were still not higher than those in Cluster B.

There were statistically significant differences between the two clusters in most laboratory indicators. However, statistically significant differences were observed between two arbitrary clusters only for AST, LDH, albumin, D-dimer and CRP, which implied that only those indicators could well-distinguish the three clusters. Few laboratory indicators, including hemoglobin, hematocrit, platelet count, and monocyte count, did not differ between any two clusters, and those indicators without any differences are not presented in Figures 3, 4.

### Clinical Prognosis of Different COVID-19 Clusters

**Figure 6:**
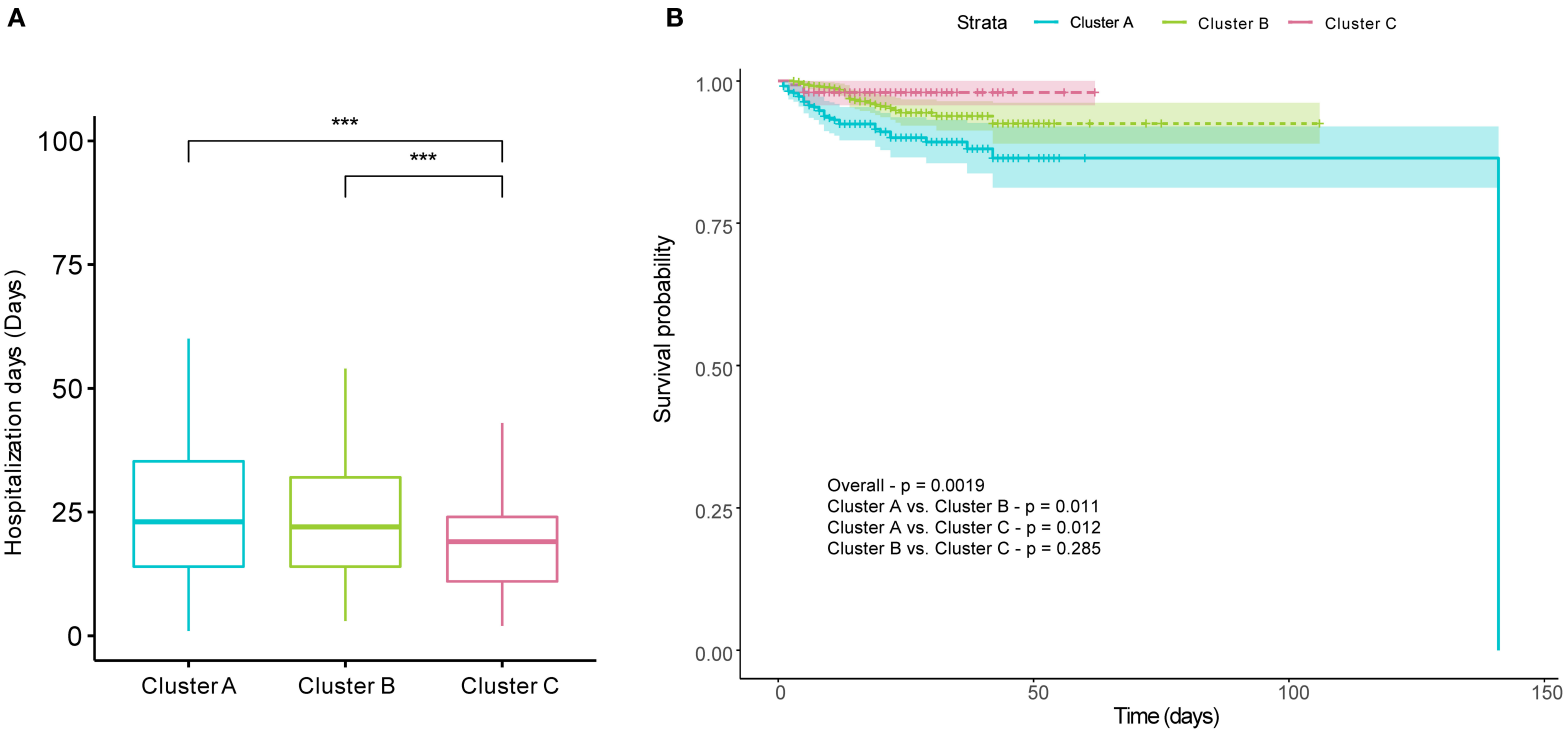
Prognosis differences of COVID-19 patients among the three clusters. There were significant differences in hospitalization days between Clusters A and C and between Clusters B and C **(A)**. **(B)** shows cumulative survival curves of three clusters comparing the survival probabilities among the three clusters, with shaded areas representing 95% confidence intervals. There were significant differences in the Kaplan–Meier survival curves between Clusters A and B, and between Clusters A and C. The Kruskal–Wallis test with Dunn's posttest and log-rank test were used to for comparisons, and *p*-values were adjusted by the Benjamini–Hochberg procedure. *p < 0.05; **p < 0.01; ***p < 0.001; ****p < 0.0001; All represents the range of adjusted *p* values in **(A)**.

To evaluate outcomes of patients in different clusters, we first compared the length of hospitalization among the three cluster first. We found that the length of hospitalization in Cluster C was lower than that in Clusters A and B (Figure 6A). Subsequently, Kaplan–Meier survival analysis of three clusters classified by FAMD-based hierarchical clustering was performed. The mortalities of the three clusters were 9.94, 4.51, and 2.01%, respectively. Survival rates were statistically assessed by the log-rank test. The results indicated that there were significant differences between Clusters A and B and between Clusters A and C (Figure 6B). As with the length of hospitalization, no difference was observed between Clusters B and C (Figure 6B).

### SVM Classifier Model Construction and Parameter Optimization

Using the results of the unsupervised hierarchical clustering, we trained an SVM classifier model to aid clinical judgement. We chose all symptoms and AST, albumin, LDH, lymphocyte count, percentage of lymphocytes, neutrophil count, percentage of eosinophils, eosinophil count, basophil count, D-dimer, PTA, INR, and CRP as predictor variables in the model. Laboratory indicators in the prediction model could well-distinguish three clusters according to the above the nonparametric Kruskal–Wallis test with Dunn's posttest, so they were chosen for the predictor variables.

**Figure 7:**
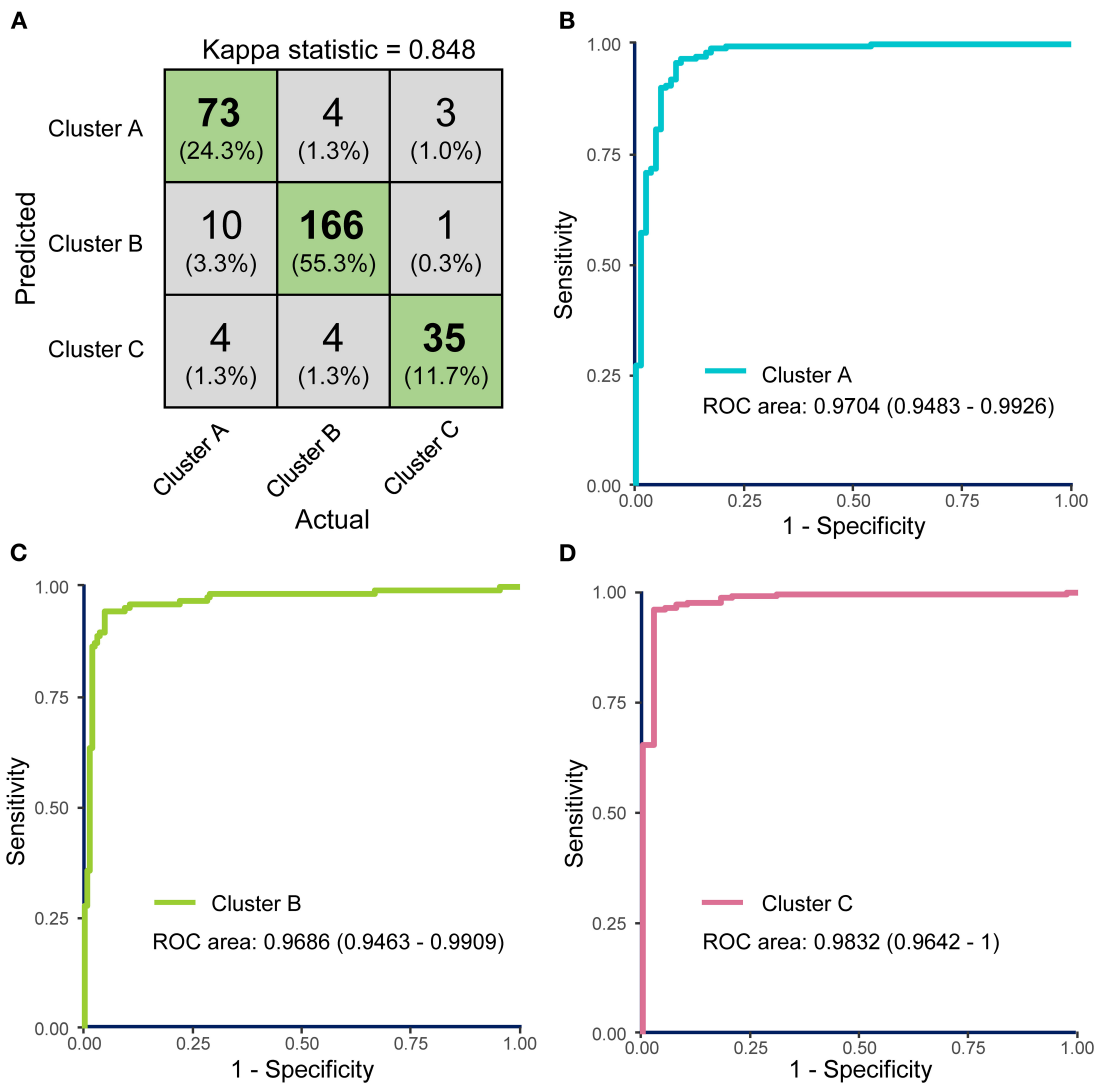
The performance of the support vector machine (SVM)-based classifier model was evaluated by receiver operating characteristics (ROC) curves. **(A)** Confusion matrix showed that most COVID-19 samples in test dataset were classified precisely. **(B–D)** ROC curves for the SVM classifier represent the predictive powers of Clusters A, B, and C, respectively. The areas under the curves (AUCs) of the three ROC curves were 0.9926 (95% CI: 0.9872–0.9981), 0.9906 (95% CI: 0.9859–0.9960), and 0.9979 (95% CI: 0.9957–1.000).

A grid-search on 10-fold cross validation for parameters was performed to find the best model, and parameters producing the best result were chosen (Supplementary Figure 5). The highest mean total kappa statistic on the training dataset was 0.847, which was predicted by the radial basis function (RBF) kernel (Supplementary Figure 5B). The confusion matrix for the classifier model on the test dataset was shown in Figure 7A, and kappa statistic of the model on the test dataset was 0.848, which suggested that the model was not over-fitted. So the RBF kernel (gamma = 0.01 and cost = 100) was chosen for the final construction of the classifier model. Three ROC curves represented the prediction performances of the three clusters respectively. The AUCs were 0.9704 (95% CI: 0.9483–0.9926), 0.9686 (95% CI: 0.9463–0.9909), and 0.9832 (95% CI: 0.9642–1), respectively (Figures 7B–D). ROC curves and AUCs also indicated the excellent predictive power of FAMD-based cluster analysis results.

Subsequently, we tested the performance of the SVM model in the case of missing data. Mean kappa statistics remained consistently >0.8, and the result indicated that the model could well-cope with up to 10% missing laboratory data imputed by KNN method (Supplementary Figure 6). Data with more than 10% missing rate could not be imputed well by KNN method, so we did not test it with higher missing rate.

There were 17 laboratory tests in the classifier model, which were not hard to get according to reviewing medical records or directly asking patients at their admission. Other thirteen predictor variables were laboratory tests, and six of them (lymphocyte count, percentage of lymphocytes, neutrophil count, eosinophil count, percentage of eosinophils, and basophil count) were belonged to routine blood tests, which is a common and cheap clinical test and easily to get. Additionally, C-reactive protein (CRP), aspartate transaminase (AST), lactic dehydrogenase (LDH), albumin, as well as D-dimer, prothrombin time activity (PTA), and international normalized ratio (INR) were commonly used to evaluate the disease progression. Even though medical institutions could not test part of them, data imputation could well-cope with this point. In short, the model has a broad range of clinical applications, and lots of predictor variables would not restrain it from application. Our classifier model is open-sourced and available at https://github.com/Spider-Rom/Support-Vector-Machine-Based-Classifier-Model-of-COVID-19-patients.

## Discussion

Over the past year, a wave of COVID-19 has hit people around the world. In response, a number of correlated studies have been carried out, and our knowledge of COVID-19 has grown rapidly. It has been reported that COVID-19 has a wide spectrum of clinical manifestations, ranging from asymptomatic carrier infection to life-threatening complications (2, 21, 22). The diversity of clinical manifestations of COVID-19 means two different things. On the one hand, the same patient could present mild symptoms shortly after infection, and the clinical manifestations could worsen as the disease progresses. On the other hand, some patients are always in asymptomatic states, but other patients might present with severe conditions. Heterogeneous clinical manifestations of COVID-19 make its diagnosis and a determination of their prognosis challenging. Moreover, a broad spectrum of COVID-19 clinical manifestations and clinical course pose difficulty in the systematic analysis of COVID-19 clinical features. Additionally, it is difficult for clinicians to give comprehensive consideration to the vast amount of information on multiple symptoms and laboratory findings, especially when patients have a less severe condition. Furthermore, the classifications of COVID-19 in past studies were often based on a few key laboratory findings or on whether complications or adverse events happened rather than based on the clinical manifestations, which would be unfavorable to systematic and comprehensive research on COVID-19.

On account of these points, FAMD-based clustering hierarchical analysis, an unsupervised machine learning method, was performed on the clinical information at the time of admission of 1,035 COVID-19 patients. The cluster analysis results in the identification of three distinct clusters: Cluster A, most severe syndromes and laboratory findings, longest hospital stays; Cluster B, intermediate severe syndromes and laboratory findings, equally long length of hospital stay with Cluster A; Cluster C, mildest clinical syndromes and laboratory findings, shortest length of hospital stays among the three clusters. Survival analysis showed that the worst survival of COVID-19 patients in Cluster A. There were no contradictions in the three clusters among laboratory findings, symptoms, and prognosis, which was also consistent with our experience in clinical practice. It is easy to see that Cluster B had the greatest number of individuals, and Cluster C had the smallest number of individuals. However, the proportion of people in each cluster could not well-reflect the proportion of each cluster within all COVID-19 patients and partly because not all infected peoples would visit hospitals.

It is not easy to follow detailed clinical features of each cluster because of too many laboratory findings and symptoms were analyzed. Overall, Cluster A had the most severe symptoms and laboratory findings among the three clusters, so Cluster A should be characterized as the “severe” cluster. Patients in Cluster B had higher levels of CRP, D-dimer, AST, and LDH, indicating more severe clinical phenotypes. However, it is interesting that Clusters B and C had significantly different frequencies of respiratory symptoms and digestive symptoms. There were higher frequencies of respiratory symptom such as cough, in Cluster B than those in Cluster C. In contrast, Cluster C almost had no respiratory symptoms. Patients in Cluster C mainly had systemic and digestive symptoms, including fever, fatigue, diarrhea, and inappetence. Remarkably, the clinical manifestations of COVID-19 patients in Cluster C were not typical due to their low frequencies of fever and cough symptoms, which may increase the difficulty of diagnosis (23).

Taken together, Clusters B and C not only represented different severity of COVID-19, but also represented different clinical disease patterns. That is, the Cluster B could be characterized as the “classical” COVID-19 cluster, and Cluster C could be characterized as the “atypical” COVID-19 cluster.

Different laboratory indicators showed different abilities to identify three clusters. LDH, an intracellular enzyme, is present in almost all human cells, and it is released to the extracellular space due to severe infections. Thus, a high level of LDH is associated with injury to the heart, lung, kidney, and other organs (24). Studies have indicated that elevated LDH levels indicate worse outcomes in COVID-19 patients (25). CRP, a well-known marker of inflammation, reflects systemic inflammation and tissue damage. Increased CRP levels are also associated with worse symptoms and worse organ injury among COVID-19 patients (26, 27). Furthermore, AST and D-dimer reflect the level of liver injury and coagulation dysfunction, respectively. These two indicators are also closely relevant to the severity of COVID-19 patients (9, 28, 29). In our study, differences were present in CRP, AST, LDH, and D-dimer between any two clusters, so these four indicators were better to distinguishing the three clusters. Clinicians should pay more attention to these indicators considering their relationship between the indicators and the disease prognosis. In addition to these four indicators, hemocyte-relevant indicators, such as lymphocytes, neutrophils, eosinophils and basophils, are linked to the severity of COVID-19 (7, 30–32). Differences in hemocyte-relevant indicators were also found among the three clusters in our study, although the ability to identify the three clusters of indicators was not as powerful as LDH and CRP. Nevertheless, abnormal hemocyte-relevant indicators of the COVID-19 patients also deserve attention.

Different frequencies of symptoms among the three clusters were also interesting, but difficult to understand. Some symptoms, such as dyspnea and diarrhea, had lower frequencies only in Cluster C; however, some other symptoms, such as sore throat, nasal obstruction or runny nose, vomiting and chills, had higher frequencies only in Cluster A. Elusive differences mean we have insufficient understanding of the disease. From the point of symptoms alone, Cluster B was closer to Cluster C than Cluster A. Contradictorily, almost all individuals in Cluster B had experienced different degrees of cough symptoms, but coughing in Cluster C was rare. Moreover, patients in Cluster C did not present cough symptoms before they were admitted to the hospital, which may indicate that patients in different clusters exhibited distinct immune response patterns when facing SARS-CoV-2 infection. It is worth noting that the three clusters did not differ in age. Therefore, we speculated that there were differences in aspects of viral loads during infection, patients' basal diseases and immune defense abilities and whether patients rested appropriately after infection. Atypical symptoms mean better outcomes; however, they are also barriers to seeking medical attention because it is difficult for the patients themselves to realize they have a viral infection. Thus, it is necessary to regularly screen high-risk individuals by pathogenic tests.

In our study, Cluster C showed the shortest hospitalization time with a median duration of 19 days. The median hospitalization days of patients in Clusters A and B was increased by 3–4 days, which means that those patients found it more difficult to recover. However, hospitalization days in our study are longer than other studies (33). Even if in Cluster C, COVID-19 patients exhibited 19 days of median length of a hospital stay. That might be associated with insufficient medical resources and inexperienced clinicians during the early COVID-19 epidemic in Wuhan.

Interestingly, there was no statistically significant difference in age or sex among the three clusters. Many studies have identified advanced age as a risk factor for adverse outcomes in COVID-19 patients (34, 35). However, three clusters divided by FAMD-based cluster analysis in our study showed significant differences in laboratory findings, symptoms, and outcomes but no significant difference in age. This might be interpreted as different disease patterns upon hospital admission may depend on the time elapsed from symptoms onset. However, the onset time of most COVID-19 patients in our study was not available. Our study only collected hospitalization days of COVID-19 patients and the time before admission was not included. Thus, it is hard to reveal the relationship between different disease patterns and the time elapsed from symptom onsets. Different lifestyles caused by gender differences may affect COVID-19-related mortality, but no difference was observed in sex among the three clusters (36). This could also be interpreted as minor discrepancy causing weak discriminative power.

We are able to view the clinical characteristics of COVID-19 from a novel perspective with the help of unsupervised learning methods. As previously mentioned, the clinical manifestation of COVID-19 is highly heterogeneous, and it is not appropriate to divide patients into several groups according to a few clinical indexes when exploring clinical characteristics of COVID-19. To obtain comprehensive and meaningful conclusions, we examined the data to ensure that patients enrolled in this study showed different condition severities. FAMD transformation was applied before cluster analysis because FAMD is a dimensionality reduction method similar to principal component analysis (PCA), which is able to handle categorical and continual variables simultaneously. Using FAMD, the dimensionality of the medical information matrix was decreased, and multicollinearity among independent variables, could make cluster analysis more effective (37). Because of the clinical diversity of samples in our study, clusters divided by FAMD-based cluster analysis have important implications for clinical practice. However, the differences among clusters were so complex and elusive that it was difficult to manually annotate the dataset. Given this, we finally constructed an SVM-based classifier model to help with cluster division. Clinicians could easily determine the severity of the illness in COVID-19 patients and propose rough prognoses with the help of the model so that the treatment schemes could be adjusted in a timely manner, especially at the time of disease outbreaks.

However, there were some unavoidable shortcomings of our study. First, some symptoms of COVID-19 patients, such as loss of taste or smell, were not included because they were hardly mentioned in the primary medical records. Second, pulmonary imaging features of COVID-19 patients were also not collected in our study. In addition, some meaningful indexes such as oxygenation index (PaO_2_/FiO_2_), blood gas test, and intensive care unit (ICU) length of stay, were not included in the study due to hard data collection. Furthermore, although asymptomatic infected individuals and mild cases individuals were enrolled, the frequencies of moderate and severely ill patients were higher than those in all COVID-19 patients in this single-center study because of selection bias considering that Tongji Hospital is not primary medical institution. Last but not least, the symptoms may not be reliable enough because of unstructured nature of the clinical records, even though we excluded parsimonious and unspecific records before analysis.

In conclusion, FAMD-based cluster analysis, an exploratory unsuspended classification method, was first applied to the clinical information at the time of admission of COVID-19 patients, and three COVID-19 clusters with different symptoms, different laboratory findings and different prognoses were identified. Our study further reveals the relationship among the symptoms, laboratory findings, and prognosis of COVID-19 from a novel perspective. Results from unsupervised hierarchical clustering also have a lot of potential to help clinicians. First, some laboratory indicators such as CRP, LDH, and AST, are crucial indexes to indicate the illness severity. It has been widely reported by other studies and our study also confirmed that. Secondly, our study demonstrated that some symptoms such as fever, dizziness, palpitations, fatigue as well as nausea and vomiting, are also important to indicating the disease severity and prognosis, which has been infrequently explored in other studies. In addition, clinicians were regularly faced with dozens of indexes including laboratory findings and symptoms. Thus, the SVM-based classifier model was constructed to aid clinical assessment, which could help with developing individualized and specific treatments for COVID-19 patients in the background of continuously increasing numbers of infected people. The prediction model can not only be used for newly admitted patients, and it also works with COVID-19 patients who were under medical treatment and need to reassess their conditions.

## Conclusions

In our study, COVID-19 patients were divided into three clusters with different clinical characteristics and prognoses using FAMD-based cluster analysis, which was the first attempt at exploratory analysis of the spectrum of COVID-19 clinical characteristics, and the relationship between clinical characteristics and outcomes of COVID-19 patients was revealed from a novel perspective. An SVM-based classifier model was constructed according to the FAMD-based cluster analysis results so that this classification based on a few key laboratory findings and symptoms of COVID-19 patients can be used conveniently in clinical practice.

## Data Availability Statement

The raw data supporting the conclusions of this article will be made available by the authors, without undue reservation.

## Ethics Statement

The studies involving human participants were reviewed and approved by the ethics committee of Tongji Hospital of Tongji Medical College of Huazhong University of Science and Technology. Written informed consent from the participants' legal guardian/next of kin was not required to participate in this study in accordance with the national legislation and the institutional requirements.

## Author Contributions

ST, ZC, and LH contributed to conception and design of the study. ZC collected the original clinical data. LH and JY per-processed the data. LH performed the statistical analysis. LH constructed and optimized the SVM model. ZC, JY, YaH, YiH, HW, YW, and ST interpreted the results. LH wrote the first draft of the manuscript. ZC, PS, XB, WL, and KQ revised the manuscript. PS and LH revised the manuscript according to the reviewer's suggestion. ZC and ST dominated and guided the revision of the manuscript. All authors have approved the submitted manuscript.

## Conflict of Interest

The authors declare that the research was conducted in the absence of any commercial or financial relationships that could be construed as a potential conflict of interest.

## Supplementary Material

Supplementary Figure 1 Distribution of missing laboratory tests in 1413 COVID-19 patients. Heatmap showed distribution of missing laboratory tests in 1413 COVID-19 patients (presence: blue; absence: white).

Supplementary Figure 2 Completeness rates of laboratory tests in 1413 COVID-19 patients. Histograms indicated the completeness rates of different laboratory tests. Only laboratory tests with above 90% completeness were kept for the next steps.

Supplementary Figure 3 Different levels of blood biochemistry tests between two clusters. **(A)** to **(K)** show the different levels of alanine transaminase (ALT), aspartate transaminase (AST), gamma-glutamyl transferase (γ-GT), alkaline phosphatase (ALP), lactic dehydrogenase (LDH), total cholesterol, urea, albumin, globin, creatinine, and estimated glomerular filtration rate (eGFR) between two clusters, respectively. **(L)** to **(X)** show the different levels of white blood cell (WBC) count, neutrophil count, lymphocyte count, eosinophil count, percentage of neutrophil, percentage of lymphocyte, percentage of eosinophil, percentage of basophil, D-dimer, prothrombin time (PT), international normalized ratio (INR), prothrombin time activity (PTA), C-reactive protein (CRP) between two clusters, respectively. The Kruskal-Wallis tests with Dunn's posttest were performed, and p value were adjusted by the Benjamini-Hochberg procedure. *, *p* < 0.05; **, *p* < 0.01; ***, *p* < 0.001; ****, *p* < 0.0001.

Supplementary Figure 4 Different levels of blood biochemistry tests among four clusters. **(A)** to **(L)** show the different levels of alanine transaminase (ALT), aspartate transaminase (AST), gamma-glutamyl transferase (γ-GT), albumin, globin, creatinine, urea, uric acid, total cholesterol (TC), blood glucose, lactic dehydrogenase (LDH) and alkaline phosphatase (ALP) among four clusters, respectively. **(M)** to **(Z)** show the different levels of white blood cell (WBC) count, percentage of lymphocyte, lymphocyte count, percentage of neutrophil, neutrophil count, percentage of eosinophil, eosinophil count, percentage of basophil, basophil count, D-dimer, prothrombin time activity (PTA), prothrombin time (PT), international normalized ratio (INR), C-reactive protein (CRP) among four clusters, respectively. The Kruskal-Wallis tests with Dunn's posttest were performed, and p value were adjusted by the Benjamini-Hochberg procedure. *, *p* < 0.05; **, *p* < 0.01; ***, *p* < 0.001; ****, *p* < 0.0001.

Supplementary Figure 5 Parameter optimization of prediction model using the cross validated grid search. Overall dataset was randomly divided into 70% training and 30% tests datasets, and the optimal parameters were found by using grid search based on 10-fold cross-validation on 70% training dataset. **(A)** Kappa statistics of the linear kernel support vector machine (SVM) with different cost value. The blue dashed line indicated the change trend of mean kappa statistics with different cost value. Grey crosses indicated different kappa statistics obtained in each ten-fold cross validation, and grey area represented the range of kappa statistics. **(B)** Heat map of mean kappa statistics of a radial basis function (RBF) kernel SVM in different cost and gamma value. **(C)** Heat map of mean kappa statistics of a polynomial kernel SVM in different degree, cost and gamma value. **(D)** Heat map of mean kappa statistics of a sigmoid kernel SVM in different cost and gamma value.

Supplementary Figure 6 Performance of prediction model in the case of missing data. Dataset was randomly divided into 50% training and 50% missing tests datasets. Missing data were imputed by *k*-nearest neighbor (KNN) method. The line chart demonstrated the distribution of kappa statistic of support vector machine in the case of about 1% to 10 % missing data. Tests were repeated with 50 times with the same missing rate. The blue dashed line indicated the change trend of mean kappa statistics with different missing rate. Grey crosses indicated different kappa statistics obtained in different tests, and grey area represented the range of kappa statistics.

Supplementary Table 1 53 independent variables (dimensions) extracted by Factor Analysis of Mixed Data.

Supplementary Table 2 Laboratory indicators and ages in Cluster A and B normal distribution test results.

Supplementary Table 3 Laboratory indicators and ages in Cluster A, B and C normal distribution test results.

Supplementary Table 4 Laboratory indicators and ages in Cluster A, B, C and D normal distribution test results.

Supplementary Table 5 Homogeneity of variance test results of albumin in two, three and four clusters.
