# Supplementary figures and images for: Exploring the Clinical Characteristics of COVID-19 Clusters Identified Using Factor Analysis of Mixed Data-Based Cluster Analysis
Source: Front Med (Lausanne). 2021 Jul 16;8:644724. doi: 10.3389/fmed.2021.644724 (PMC8323882; doi:10.3389/fmed.2021.644724)

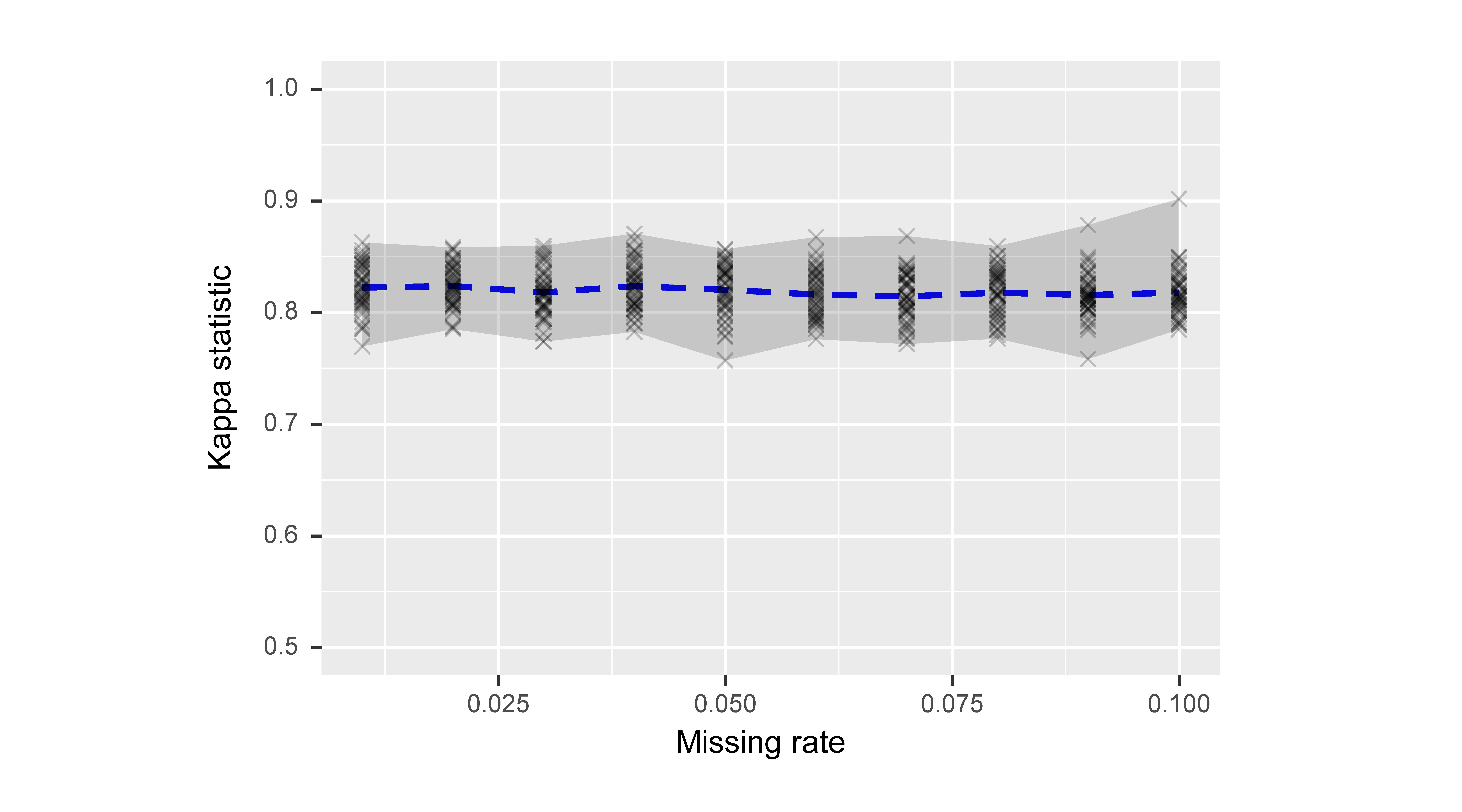

Supplement: Supplementary Figure 6 — Performance of prediction model in the case of missing data. Dataset was randomly divided into 50% training and 50% missing tests datasets. Missing data were imputed by k-nearest neighbor (KNN) method. The line chart demonstrated the distribution of kappa statistic of support vector machine in the case of about 1% to 10 % missing data. Tests were repeated with 50 times with the same missing rate. The blue dashed line indicated the change trend of mean kappa statistics with different missing rate. Grey crosses indicated different kappa statistics obtained in different tests, and grey area represented the range of kappa statistics. [file Image_6.TIF]
