# Supplementary material for: Exploring the Clinical Characteristics of COVID-19 Clusters Identified Using Factor Analysis of Mixed Data-Based Cluster Analysis
Source: Front Med (Lausanne). 2021 Jul 16;8:644724. doi: 10.3389/fmed.2021.644724 (PMC8323882; doi:10.3389/fmed.2021.644724)
Supplement: Supplementary Table 1 — 53 independent variables (dimensions) extracted by Factor Analysis of Mixed Data. [file Table_1.DOCX]

## Supplementary Tables

Supplementary Table 1. 53 independent variables (dimensions) extracted by Factor Analysis of Mixed Data.

| Dimension | Eigenvalue | Variance percent | Cumulative variance percent |
| --- | --- | --- | --- |
| Dim.1 | 7.67 | 14.48% | 14.48% |
| Dim.2 | 3.78 | 7.13% | 21.61% |
| Dim.3 | 3.44 | 6.50% | 28.11% |
| Dim.4 | 2.49 | 4.69% | 32.80% |
| Dim.5 | 2.47 | 4.66% | 37.46% |
| Dim.6 | 2.22 | 4.18% | 41.65% |
| Dim.7 | 1.94 | 3.66% | 45.30% |
| Dim.8 | 1.64 | 3.09% | 48.40% |
| Dim.9 | 1.63 | 3.08% | 51.47% |
| Dim.10 | 1.41 | 2.66% | 54.13% |
| Dim.11 | 1.30 | 2.46% | 56.60% |
| Dim.12 | 1.26 | 2.37% | 58.96% |
| Dim.13 | 1.19 | 2.25% | 61.21% |
| Dim.14 | 1.14 | 2.14% | 63.36% |
| Dim.15 | 1.13 | 2.13% | 65.49% |
| Dim.16 | 1.05 | 1.99% | 67.48% |
| Dim.17 | 0.98 | 1.86% | 69.33% |
| Dim.18 | 0.96 | 1.81% | 71.15% |
| Dim.19 | 0.94 | 1.78% | 72.92% |
| Dim.20 | 0.91 | 1.72% | 74.65% |
| Dim.21 | 0.88 | 1.66% | 76.30% |
| Dim.22 | 0.87 | 1.63% | 77.93% |
| Dim.23 | 0.84 | 1.58% | 79.51% |
| Dim.24 | 0.80 | 1.50% | 81.01% |
| Dim.25 | 0.77 | 1.46% | 82.47% |
| Dim.26 | 0.77 | 1.45% | 83.92% |
| Dim.27 | 0.76 | 1.44% | 85.36% |
| Dim.28 | 0.70 | 1.33% | 86.68% |
| Dim.29 | 0.69 | 1.30% | 87.99% |
| Dim.30 | 0.66 | 1.24% | 89.22% |
| Dim.31 | 0.64 | 1.20% | 90.42% |
| Dim.32 | 0.61 | 1.16% | 91.58% |
| Dim.33 | 0.59 | 1.11% | 92.69% |
| Dim.34 | 0.55 | 1.03% | 93.73% |
| Dim.35 | 0.47 | 0.90% | 94.62% |
| Dim.36 | 0.46 | 0.87% | 95.50% |
| Dim.37 | 0.41 | 0.77% | 96.27% |
| Dim.38 | 0.38 | 0.72% | 96.99% |
| Dim.39 | 0.32 | 0.62% | 97.60% |
| Dim.40 | 0.26 | 0.49% | 98.10% |
| Dim.41 | 0.20 | 0.38% | 98.48% |
| Dim.42 | 0.18 | 0.34% | 98.82% |
| Dim.43 | 0.17 | 0.32% | 99.14% |
| Dim.44 | 0.15 | 0.29% | 99.43% |
| Dim.45 | 0.13 | 0.25% | 99.68% |
| Dim.46 | 0.05 | 0.10% | 99.79% |
| Dim.47 | 0.05 | 0.09% | 99.87% |
| Dim.48 | 0.04 | 0.08% | 99.94% |
| Dim.49 | 0.02 | 0.05% | 100.00% |
| Dim.50 | <0.01 | <0.01% | 100.00% |
| Dim.51 | <0.01 | <0.01% | 100.00% |
| Dim.52 | <0.01 | <0.01% | 100.00% |
| Dim.53 | <0.01 | <0.01% | 100.00% |

Dim: dimension. Dimensions are arranged in order from large to small eigenvalue.

Supplementary Table 2. Laboratory indicators and ages in Cluster A and B normal distribution test results

| Laboratory tests | Cluster A | Cluster B |
| --- | --- | --- |
| ALT (U/L) | <0.00001 | <0.00001 |
| AST (U/L) | <0.00001 | <0.00001 |
| γ-GT (U/L) | <0.00001 | <0.00001 |
| Albumin (g/L) | 0.12908 | 0.05942 |
| Globulin (g/L) | 0.00059 | <0.00001 |
| Total protein (g/L) | 0.00024 | 0.09719 |
| Creatinine (μmol/L) | <0.00001 | <0.00001 |
| Urea (mmol/L) | <0.00001 | <0.00001 |
| Uric acid (μmol/L) | <0.00001 | <0.00001 |
| Total cholesterol (mmol/L) | 0.00246 | <0.00001 |
| Blood glucose (mmol/L) | <0.00001 | <0.00001 |
| LDH (U/L) | <0.00001 | <0.00001 |
| ALP (U/L) | <0.00001 | <0.00001 |
| WBC count (×10^9^/L) | <0.00001 | <0.00001 |
| RBC count (×10^12^/L) | 0.00001 | 0.01698 |
| Lymphocyte rate (%) | 0.00004 | 0.00001 |
| Lymphocyte count (×10^9^/L) | <0.00001 | <0.00001 |
| Monocyte rate (%) | <0.00001 | <0.00001 |
| Monocyte count (×10^9^/L) | <0.00001 | <0.00001 |
| Neutrophil rate (%) | 0.00031 | 0.07052 |
| Neutrophil count (×10^9^/L) | <0.00001 | <0.00001 |
| Eosinophil rate (%) | <0.00001 | 0.00013 |
| Eosinophil count (×10^9^/L) | <0.00001 | <0.00001 |
| Basophil rate (%) | <0.00001 | <0.00001 |
| Basophil count (×10^9^/L) | <0.00001 | <0.00001 |
| Hematocrit (%) | <0.00001 | <0.00001 |
| Hemoglobin (g/L) | <0.00001 | <0.00001 |
| Platelet (×10^9^/L) | <0.00001 | <0.00001 |
| D-dimer (μg/ml FEU) | <0.00001 | <0.00001 |
| PTA (%) | <0.00001 | <0.00001 |
| PT (s) | <0.00001 | <0.00001 |
| INR | <0.00001 | <0.00001 |
| CRP (mg/L) | <0.00001 | <0.00001 |
| eGFR (ml/min/1.73 m^2^) | <0.00001 | <0.00001 |
| Age (year) | 0.00001 | <0.00001 |

ALT: alanine transaminase; AST: aspartate transaminase; γ-GT: gamma-glutamyl transferase; LDH: lactic dehydrogenase; ALP: alkaline phosphatase; WBC: white blood cell; RBC: red blood cell. PTA: prothrombin time activity; PT: prothrombin time; INR: international normalized ratio; CRP: C-reactive protein; eGFR: estimated glomerular filtration rate. Laboratory tests are presented as median (interquartile ranges). P value were calculated by Shapiro-Wilk test via function shapiro.test() in R.

Supplementary Table 3. Laboratory indicators and ages in Cluster A, B and C normal distribution test results

| Laboratory tests | Cluster A | Cluster B | Cluster C |
| --- | --- | --- | --- |
| ALT (U/L) | <0.00001 | <0.00001 | <0.00001 |
| AST (U/L) | <0.00001 | <0.00001 | <0.00001 |
| γ-GT (U/L) | <0.00001 | <0.00001 | <0.00001 |
| Albumin (g/L) | 0.12908 | 0.14740 | 0.63248 |
| Globulin (g/L) | 0.00059 | <0.00001 | 0.29768 |
| Total protein (g/L) | 0.00024 | 0.12343 | 0.50298 |
| Creatinine (μmol/L) | <0.00001 | <0.00001 | <0.00001 |
| Urea (mmol/L) | <0.00001 | <0.00001 | 0.00080 |
| Uric acid (μmol/L) | <0.00001 | <0.00001 | 0.01529 |
| Total cholesterol (mmol/L) | 0.00246 | <0.00001 | 0.00785 |
| Blood glucose (mmol/L) | <0.00001 | <0.00001 | <0.00001 |
| LDH (U/L) | <0.00001 | <0.00001 | <0.00001 |
| ALP (U/L) | <0.00001 | <0.00001 | 0.00004 |
| WBC count (×10^9^/L) | <0.00001 | <0.00001 | 0.00010 |
| RBC count (×10^12^/L) | 0.00001 | 0.02066 | 0.41678 |
| Lymphocyte rate (%) | 0.00004 | 0.00003 | 0.03695 |
| Lymphocyte count (×10^9^/L) | <0.00001 | <0.00001 | 0.00002 |
| Monocyte rate (%) | <0.00001 | <0.00001 | 0.21158 |
| Monocyte count (×10^9^/L) | <0.00001 | <0.00001 | 0.21131 |
| Neutrophil rate (%) | 0.00031 | 0.05758 | 0.60507 |
| Neutrophil count (×10^9^/L) | <0.00001 | <0.00001 | <0.00001 |
| Eosinophil rate (%) | <0.00001 | 0.00146 | 0.03526 |
| Eosinophil count (×10^9^/L) | <0.00001 | <0.00001 | <0.00001 |
| Basophil rate (%) | <0.00001 | <0.00001 | <0.00001 |
| Basophil count (×10^9^/L) | <0.00001 | <0.00001 | <0.00001 |
| Hematocrit (%) | <0.00001 | <0.00001 | <0.00001 |
| Hemoglobin (g/L) | <0.00001 | <0.00001 | <0.00001 |
| Platelet (×10^9^/L) | <0.00001 | <0.00001 | 0.00120 |
| D-dimer (μg/ml FEU) | <0.00001 | <0.00001 | <0.00001 |
| PTA (%) | <0.00001 | <0.00001 | 0.08300 |
| PT (s) | <0.00001 | <0.00001 | <0.00001 |
| INR | <0.00001 | <0.00001 | <0.00001 |
| CRP (mg/L) | <0.00001 | <0.00001 | <0.00001 |
| eGFR (ml/min/1.73 m^2^) | <0.00001 | <0.00001 | 0.06137 |
| Age (year) | 0.00001 | <0.00001 | 0.00420 |

ALT: alanine transaminase; AST: aspartate transaminase; γ-GT: gamma-glutamyl transferase; LDH: lactic dehydrogenase; ALP: alkaline phosphatase; WBC: white blood cell; RBC: red blood cell. PTA: prothrombin time activity; PT: prothrombin time; INR: international normalized ratio; CRP: C-reactive protein; eGFR: estimated glomerular filtration rate. Laboratory tests are presented as median (interquartile ranges). P value were calculated by Shapiro-Wilk test via function shapiro.test() in R.

Supplementary Table 4. Laboratory indicators and ages in Cluster A, B, C and D normal distribution test results

| Laboratory tests | Cluster A | Cluster B | Cluster C | Cluster D |
| --- | --- | --- | --- | --- |
| ALT (U/L) | <0.00001 | <0.00001 | <0.00001 | <0.00001 |
| AST (U/L) | <0.00001 | <0.00001 | <0.00001 | <0.00001 |
| γ-GT (U/L) | <0.00001 | <0.00001 | <0.00001 | <0.00001 |
| Albumin (g/L) | 0.18719 | 0.14740 | 0.63248 | 0.54806 |
| Globulin (g/L) | 0.00521 | <0.00001 | 0.29768 | 0.09481 |
| Total protein (g/L) | 0.00050 | 0.12343 | 0.50298 | 0.99723 |
| Creatinine (μmol/L) | <0.00001 | <0.00001 | <0.00001 | 0.02175 |
| Urea (mmol/L) | <0.00001 | <0.00001 | 0.00080 | <0.00001 |
| Uric acid (μmol/L) | <0.00001 | <0.00001 | 0.01529 | 0.00084 |
| Total cholesterol (mmol/L) | 0.02739 | <0.00001 | 0.00785 | 0.00623 |
| Blood glucose (mmol/L) | <0.00001 | <0.00001 | <0.00001 | <0.00001 |
| LDH (U/L) | <0.00001 | <0.00001 | <0.00001 | 0.00002 |
| ALP (U/L) | <0.00001 | <0.00001 | 0.00004 | 0.00002 |
| WBC count (×10^9^/L) | <0.00001 | <0.00001 | 0.00010 | 0.00007 |
| RBC count (×10^12^/L) | <0.00001 | 0.02066 | 0.41678 | 0.07083 |
| Lymphocyte rate (%) | 0.00006 | 0.00003 | 0.03695 | 0.56602 |
| Lymphocyte count (×10^9^/L) | <0.00001 | <0.00001 | 0.00002 | 0.52001 |
| Monocyte rate (%) | <0.00001 | <0.00001 | 0.21158 | 0.00585 |
| Monocyte count (×10^9^/L) | <0.00001 | <0.00001 | 0.21131 | 0.01625 |
| Neutrophil rate (%) | 0.00023 | 0.05758 | 0.60507 | 0.87004 |
| Neutrophil count (×10^9^/L) | <0.00001 | <0.00001 | <0.00001 | <0.00001 |
| Eosinophil rate (%) | <0.00001 | 0.00146 | 0.03526 | 0.10853 |
| Eosinophil count (×10^9^/L) | <0.00001 | <0.00001 | <0.00001 | 0.00005 |
| Basophil rate (%) | <0.00001 | <0.00001 | <0.00001 | <0.00001 |
| Basophil count (×10^9^/L) | <0.00001 | <0.00001 | <0.00001 | 0.00009 |
| Hematocrit (%) | <0.00001 | <0.00001 | <0.00001 | 0.00003 |
| Hemoglobin (g/L) | <0.00001 | <0.00001 | <0.00001 | 0.24127 |
| Platelet (×10^9^/L) | <0.00001 | <0.00001 | 0.00120 | 0.12030 |
| D-dimer (μg/ml FEU) | <0.00001 | <0.00001 | <0.00001 | <0.00001 |
| PTA (%) | <0.00001 | <0.00001 | 0.08300 | 0.18935 |
| PT (s) | <0.00001 | <0.00001 | <0.00001 | 0.04977 |
| INR | <0.00001 | <0.00001 | <0.00001 | 0.01872 |
| CRP (mg/L) | <0.00001 | <0.00001 | <0.00001 | <0.00001 |
| eGFR (ml/min/1.73 m^2^) | <0.00001 | <0.00001 | 0.06137 | 0.03367 |
| Age (year) | 0.00001 | <0.00001 | 0.00420 | 0.60911 |

ALT: alanine transaminase; AST: aspartate transaminase; γ-GT: gamma-glutamyl transferase; LDH: lactic dehydrogenase; ALP: alkaline phosphatase; WBC: white blood cell; RBC: red blood cell. PTA: prothrombin time activity; PT: prothrombin time; INR: international normalized ratio; CRP: C-reactive protein; eGFR: estimated glomerular filtration rate. Laboratory tests are presented as median (interquartile ranges). P value were calculated by Shapiro-Wilk test via function shapiro.test() in R.

Supplementary Table 5. Homogeneity of variance test results of albumin in two, three and four clusters

|  | Bartlett's K-squared | Degree of freedom | p Value |
| --- | --- | --- | --- |
| Two clusters | 10.677 | 1 | 0.00109 |
| Three clusters | 11.231 | 2 | 0.00364 |
| Four clusters | 12.458 | 3 | 0.00597 |

P value were calculated by Bartlett's Test via function bartlett.test() in R.
